# Association between Plasma Antibody Response and Protection in Rainbow Trout *Oncorhynchus mykiss* Immersion Vaccinated against *Yersinia ruckeri*

**DOI:** 10.1371/journal.pone.0018832

**Published:** 2011-06-24

**Authors:** Martin K. Raida, Jørgen Nylén, Lars Holten-Andersen, Kurt Buchmann

**Affiliations:** 1 Department of Veterinary Disease Biology, Faculty of Life Sciences, University of Copenhagen, Frederiksberg, Denmark; 2 Intervet/Schering-Plough Animal Health, Ballerup, Denmark; Centre de Recherche Public de la Santé (CRP-Santé), Luxembourg

## Abstract

A key hallmark of the vertebrate adaptive immune system is the generation of antigen-specific antibodies from B cells. Fish are the most primitive gnathostomes (jawed vertebrates) possessing an adaptive immune system. Vaccination of rainbow trout against enteric redmouth disease (ERM) by immersion in *Yersinia ruckeri* bacterin confers a high degree of protection to the fish. The immune mechanisms responsible for protection may comprise both cellular and humoral elements but the role of specific immunoglobulins in this system has been questioned and not previously described. The present study demonstrates significant increase in plasma antibody titers following immersion vaccination and significantly reduced mortality during *Y. ruckeri* challenge.

Rainbow trout were immersion-vaccinated, using either a commercial ERM vaccine (AquaVac™ ERM vet) or an experimental *Y. ruckeri* bacterin. Half of the trout vaccinated with AquaVac™ ERM vet received an oral booster (AquaVac™ ERM Oral vet). Sub-groups of the fish from each group were subsequently exposed to 1x10^9^ CFU *Y. ruckeri*/ml either eight or twenty-six weeks post vaccination (wpv). All vaccinated groups showed 0% mortality when challenged, which was highly significant compared to the non-vaccinated controls (40 and 28% mortality eight and twenty-six weeks post vaccination (wpv), respectively) (P<0.0001). Plasma samples from all groups of vaccinated fish were taken 0, 4, 8, 12, 16 and 26 wpv. and *Y. ruckeri* specific IgM antibody levels were measured with ELISA. A significant increase in titers was recorded in vaccinated fish, which also showed a reduced bacteremia during challenge. *In vitro* plasma studies showed a significantly increased bactericidal effect of fresh plasma from vaccinated fish indicating that plasma proteins may play a role in protection of vaccinated rainbow trout.

## Introduction

*Yersinia ruckeri* is the aetiological agent of enteric red mouth (ERM) disease or yersiniosis, affecting salmonids in general and rainbow trout in particular [1], [2]. Although generally well controlled by means of vaccination and antibiotic treatment, this disease is still causing outbreaks in all trout-producing countries worldwide [3]. In some cases the losses due to this disease can be as high as 30–70% of the stock [4]. Protective immunity in rainbow trout against ERM induced by immersion vaccination using formalin killed *Y. ruckeri* has been known since 1976. The method meets the requirements of the trout farming industry and their call for easily handled vaccination techniques, high through-put of animals in a short time, a low stress-induction, a good protection and high safety [5], [6]. *Y. ruckeri* bacterin can be administrated by intraperitoneal (i.p.) injection, immersion and oral administration [7] and the obtained protective immunity is superior with i.p. injection followed by immersion, and oral administration [7]. The explanation for this observation might be that the protective effect of the bacterin seem to be dependent on the amount of bacterin uptake in the rainbow trout [8]. In salmonids gill epithelial cells have been shown to be an important site for bacterin uptake following immersion vaccination [9], [10]. It has been demonstrated that the duration of protective immunity depends on the bacterin concentration, length of immersion time, antigen uptake and the size and species of fish [11]. However the immunological mechanism behind the protective effect of the ERM immersion vaccination is still not fully described [6]. It has been reported that antibodies in rainbow trout only in few cases are associated with protection following immersion vaccination [12] and protection induced by i.p. injection of *Y. ruckeri* bacterin does not seem to be due to agglutinating antibodies [13], [14]. A range of genes encoding immune relevant effector molecules are known to be activated in the spleen of ERM immersion vaccinated rainbow trout fry, indicating activation of a systemic immune response [15]. Since *Y. ruckeri* is primarily an extracellular pathogen, and immersion vaccinated rainbow trout are protected against ERM for a least one year [16] is it likely that specific antibodies are among the protective mechanism. The purpose of the present study is to investigate whether there is an association between production of specific antibodies against *Y. ruckeri* and the protection in immersion vaccinated rainbow trout. Further, the effect of an oral booster vaccination following a primary immersion vaccination was evaluated.

## Materials and Methods

### Fish and rearing conditions

Rainbow trout (Skinderup strain from Jutland, Denmark) were hatched and reared under pathogen-free conditions (Danish Centre for Wild Salmon, Randers, Denmark). The pathogen-free status was achieved by introducing certified disinfected eggs to the recirculated system. Fish were brought to the experimental fish keeping facility at the University of Copenhagen when reaching an average body weight of 25±3 g. The pathogen-free status of the fish was confirmed by standard bacteriological and parasitological techniques upon their arrival in the laboratory. To confirm that fish were sero-negative for *Y. ruckeri* blood samples for specific ELISA-tests were taken regularly from the same batch of fish before experimental start (data not shown).

**Figure 1:**
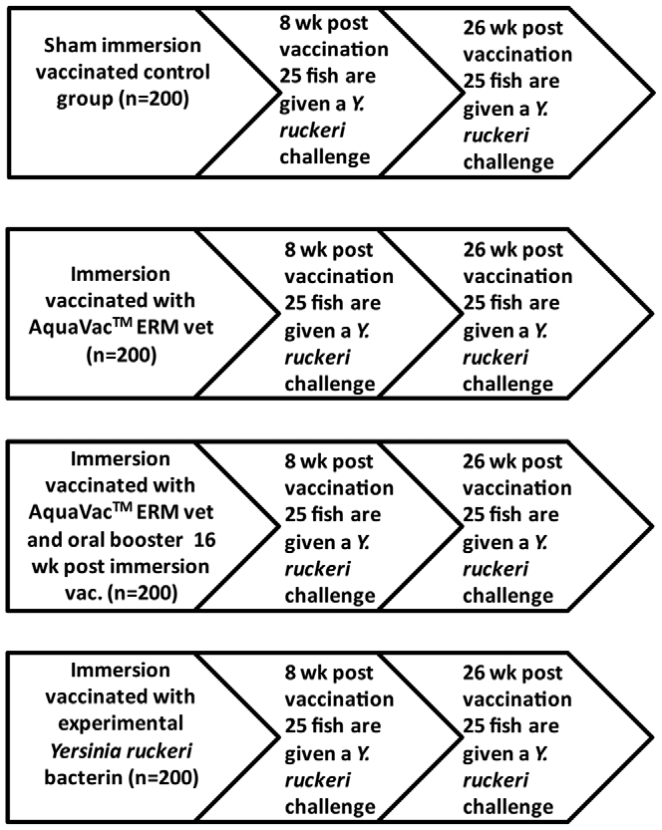
Flow chart of the experimental setup. A total of 800 rainbow trout were divided into four groups each containing 200 fish. One group was immersion vaccinated with the experimental bacterin vaccine. Two groups were immersion-vaccinated with the commercial AquaVac™ ERM. One of these groups received an oral booster vaccination with AquaVac™ ERM Oral vet 16 weeks post vaccination. All vaccines were diluted 1∶10 in water, and the fish were immersed for 5 minutes. The control group was sham-immersion vaccinated in pure water. A subsample of 25 rainbow trout from each group were given a challenge with *Y. ruckeri* 8 and 26 weeks post vaccination to monitor protection. Ten fish from each group were killed and used for plasma sampling 4, 8, 12, 16, and 26 weeks post vaccination.

The 800 fish were kept in four 120 L tanks (Fig. 1) with bio-filters (Eheim, Germany) and maintained at a 12 h light and 12 h dark cycle in aerated (100% oxygen saturation) tap water at 13°C. They were fed a commercial trout feed (BioMar, Denmark) (1% biomass per day). All procedures were conducted in accordance with the regulations set forward by the Danish Ministry of Justice and animal protection committees by Danish Animal Experiments Inspectorate permit 2006/561-1302 and in compliance with European Community Directive 86/609. The present study were approved and controlled by our institutional review board with the FELASA accreditation No 006/03/28.

### Vaccines

Three vaccines were used in the present experiment (Fig. 1). One was an experimental immersion vaccine consisting of a bacterin of 5×10^9^ formalin killed *Y. ruckeri*/ml. The strain used was serotype O1, biotype 1, with confirmed virulence for rainbow trout [15], [17], [18], [19]. The bacteria were grown in LB-medium (Oxoid LP0042, Tryptone 10 g, Oxoid LP0021Yeast-extract 5 g, NaCl 5 g, H_2_O to 1000 ml, pH 7.4) at 20°C for 36 h and enumerated as colony forming units (CFU) by the spread plate method on blood agar (Blood agar base CM55 [Oxoid] supplemented with 5% bovine blood). *Y. ruckeri* were killed by adding 1% formaldehyde to the culture and subsequently incubating for 2 hours on a plate shaker. The killed bacteria were washed 3 times with PBS and the effect of formaldehyde killing was verified by plate spreading onto blood agar.

The second immersion vaccine used was the commercial AquaVac™ ERM (Intervet/Schering-Plough Animal Health) containing 5×10^9^ CFU/ml formalin killed *Y. ruckeri* (Hagerman strain, serotype O1, biotype 1). The third vaccine used was the oral booster vaccine AquaVac™ ERM Oral vet (Intervet/Schering-Plough Animal Health) containing 5×10^8^ CFU/ml formalin killed *Y. ruckeri* (Hagerman strain, serotype O1, biotype 1).

### Vaccination

A total of 800 rainbow trout were divided into four groups each containing 200 fish (Fig. 1). One group of 200 rainbow trout were immersion vaccinated with the experimental vaccine. Two groups were immersion-vaccinated with the commercial AquaVac™ ERM. One of these groups received an oral booster vaccination with AquaVac™ ERM Oral vet 16 weeks post immersion vaccination. The oral booster vaccine was coated onto the feed pellets according to the manufacturer's recommendations (Intervet/Schering-Plough Animal Health). Each fish received 0.01 ml oral vaccine in feed pr. day from day 1–5, then normal feed without vaccine from day 6–10, and finally vaccine coated feed from day 11–15. All immersion vaccines were diluted 1∶10 in water, and the fish were immersed for 5 minutes. The control groups were sham-bath vaccinated in pure water.

### Blood sampling

Ten fish from each group were sampled 0, 4, 8, 12, 16 and 26 weeks post primary vaccination. Fish were killed by an overdose of MS222 (100 mg/l) (Sigma-Aldrich, Denmark), and blood was sampled from *vena caudalis* using heparinised syringes and immediately centrifuged at 4000 x g for 5 min (4°C), where-upon plasma was recovered and stored (−20°C).

### ELISA for determination of *Y. ruckeri* specific IgM antibodies

Enzyme-linked immunosorbent assay (ELISA) was used to detect the presence of *Y. ruckeri* O1 specific IgM in plasma. For coating, each well of the microplates (flat-bottom 96-well plates, MaxiSorp™, Nunc) was filled with 100 µL coating buffer (50 mM carbonate buffer, pH 9.6, C-3041, Sigma) containing 5 µg/mL of antigen (sonically disrupted *Y. ruckeri*, same isolate as used for experimental immersion vaccine and challenge experiments) and incubated overnight at 4°C. After removal of coating solution, the unbound antigen was removed by three washes with 400 µL washing buffer (0.1% Tween-20 in phosphate buffered saline (PBS; pH 7.2)). Blocking of free binding sites was performed by 1 h incubation with 150 µl blocking buffer (1% bovine serum albumin (BSA) in washing buffer). After blocking, microplates were washed three times, aspirated, sealed (micro plate seal, Nunc) and stored at −20°C until use.

The optimal ratio between specific reaction and background binding was found as a 50 fold dilution of plasma in pilot experiments (data not shown). Therefore, 50 fold dilutions of plasma with assay diluents buffer (PBS with 0.5% Tween 20) were made in triplicate from each fish, whereupon plasma samples were added to the wells, sealed and incubated at 4°C overnight.

Microplates were then washed three times and 50 µL of a mouse anti-salmonid Ig antibody solution (MCA2182, AbD serotec, 1∶400 dilution in assay buffer) was added. After 1 h incubation at room temperature, the micro plates were washed three times, and 50 µl were added to each well of a Fab'-HRP solution (STAR13B, AbD serotech, 1∶400 dilution in assay buffer). Optimal concentrations of the commercial antibodies were established by chessboard titration experiments (data not shown). Microplates were then incubated for additional 1 h at room temperature followed by five washes. Substrate solution was added (100 µl/well of TMB, Sigma) and after 10 min a stop solution (100 µl 1N HCl/well) was added, and the absorbance was read at 450 (PowerWave 340, BioTek). The limit of detection for the assay was set as the concentration corresponding to the signal three standard deviations (0.002) above the mean for blank wells (0.049) (sample dilution buffer only).

Wells with all antibodies and substrates except sample material were included as negative controls for the ELISA and this background OD value was subtracted from all samples. A positive reference control serum sample, taken from a rainbow trout which were ip. vaccinated tree times with *Yersinia ruckeri* bacterin in Freunds's incomplete adjuvant, was run in triplicate at each plate and used as inter-plate calibrator.

### Production of high titer reference sera

The injected vaccine contained an 48 h culture of *Y. ruckeri* O1 which were inactivated with 0.9% formaldehyde for 2 h at room temperature, washed with PBS, adjusted to an optical density corresponding to 2×10^8^ cells/mL PBS, and emulsified with an equal volume of Freund's incomplete adjuvant (FIA; Sigma, St. Louis, Mo.). The fish was immunised by intraperitoneal injections with 0.1 mL of formalin-killed bacterial vaccine suspension (1×10^7^ cells/injection). Immunisations were given three times with 500 day degree intervals (six weeks).

### Challenge experiments

Challenge were conducted using 1×10^9^ CFU *Y. ruckeri*/ml in water for 1 h (corresponding to a previously determined LD_50_ for rainbow trout at this size (data not shown), where after fish were transferred to clean water in the fish tanks. All immersion vaccinated and control groups were challenged as described. All fish that survived the 28 days of challenge were killed by an overdose of MS222 (100 mg/l).

### Isolation of *Y. ruckeri* in fish head kidney following challenge

Bacterial samples from the head kidney from all fish that died following challenge were cultured on blood agar plates. Mortalities were only considered to be caused by *Y. ruckeri* if this specific bacteria species was recovered as pure culture from the head kidney.

### Detection of *Y. ruckeri* in blood *in vivo* post-infection

Blood were collected from 5 fish (killed with an overdose of MS-222, 100 mg/l) from each of the four groups 3, 7 and 14 days post infection, and the number of CFU/ml *Y. ruckeri* from each fish were determined by plate spreading onto blood agar in a 10-fold dilution series (in triplicate) as previously described [18].

### Bactericidal effect of plasma *in vitro*

In order to investigate whether plasma from immersion vaccinated rainbow trout contained increased amounts of humoral factors with ability to kill *Y. ruckeri* an *in vitro* test was performed using plasma from fish sampled at 8 and 26 wpv. A 60 µl plasma aliquot from each fish (n = 10) was mixed 1∶1 with *Y. ruckeri* (5,6×10^3^ CFU/ml in PBS) in a round-bottom 96-well microtiter plate. After 1 h. at 20°C a volume of 10 µl of the solution was plated onto blood agar in triplicate. A plasma sample without bacteria served as a negative control and as a positive control *Y. ruckeri* was incubated in PBS without plasma. Heat inactivated trout plasma samples were included as well but resulted in excessively *Y. ruckeri* overgrown agar plates (data not shown).

### Calculations and statistical analysis

Results from the challenge experiments were analyzed using the Kaplan-Meier test, which were used to analyse for differences in mortality between groups. Kruskal-Wallis test were used to compare the amount of bacteria in the blood and the bactericidal effect of plasma between the different groups. ANOVA with Tukey post test were used to analyse the ELISA results. All statistical tests were carried out using GraphPad Prism 4 (GraphPad Software, Inc. San Diego, USA) and a significance level of 5% was applied in all tests.

## Results

### Challenge experiments

**Figure 2:**
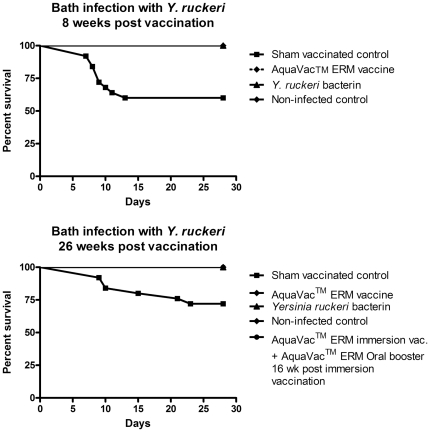
Protective effect of immersion vaccines following bath challenge. A). Percentage survival of *Y. ruckeri* bath infected rainbow trout (n = 25 in each group) eight weeks post immersion vaccination and B) twenty-six weeks post immersion vaccination. All immersion vaccinated groups of rainbow trout showed a survival rate of 100% during bath challenge with 1×10^9^ CFU/ml *Y. ruckeri* 8 and 26 weeks post vaccination, which is significantly higher survival rate than the sham-vaccinated control group (p<0.0001). The mortality in the sham vaccinated control group was 40% (A) and 28% (B), 8 and 26 weeks, respectively.

Subsamples of rainbow trout from all immersion vaccinated groups of rainbow trout showed 0% mortality following bath challenge with 1×10^9^ CFU/ml *Y. ruckeri* 8 and 26 wpv. All immersion vaccinated groups had significantly lower mortality rates than the sham-vaccinated control group (p<0.0001) (Fig. 2). The mortality in the sham-vaccinated control group was 40% and 28%, 8 and 26 weeks post sham-vaccination, respectively.

### Re-isolation of pathogen

*Y. ruckeri* was re-isolated from the head kidney of all fish that died following challenge. Dead fish exhibited external signs associated with ERM infection including petechial haemorrhages in the mouth, around the anus and at the basis of the dorsal fins.

### Detection of *Y. ruckeri* specific IgM antibodies in plasma

The *Y. ruckeri* specific IgM antibody titers were significantly higher 26 weeks post-vaccination compared to week 4 post-vaccination. In the group immersion vaccinated with the *Y. ruckeri* bacterin the titer 26 weeks post vaccination was significantly higher than the titer obtained 4, 8 and 12 weeks post-vaccination (P<0.001, P<0.05 and P<0.05) respectively. Titers in weeks 4 and 8 were significantly higher in the group which was immersion vaccinated with AquaVac™ ERM compared to the fish which were immersion vaccinated in bacterin (P<0.05).

### Detection of *Y. ruckeri* in blood post challenge

The presence of *Y. ruckeri* in the blood was detected in infected sham-vaccinated trout at all sampling points post-infection. Three days post infection (dpi.) *Y. ruckeri* was only detected in the blood of fish from the sham-vaccinated group. 7 dpi. bacteria were also detected in the AquaVac™ ERM vaccinated-group. The absence of *Y. ruckeri* 7 dpi. in the blood of the bacterin vaccinated group is highly significant relative to the non vaccinated group (p<0.01). The CFU/ml blood of *Y. ruckeri* increased in the non-vaccinated control group between each sampling (p<0.001). The CFU/ml of *Y. ruckeri* in the blood of sham-vaccinated fish was significantly higher 14 dpi.compared to both the experimental bacterin and AquaVac™ ERM immersion vaccinated group (p<0.001 and p<0.05). No significant difference was detected between the two vaccinated groups (Fig. 4).

### Bactericidal effect of plasma

Plasma from both sham-vaccinated and vaccinated trout contained bactericidal factors (Fig. 5) but it was shown that plasma obtained from trout immersion vaccinated with *Y. ruckeri* bacterin and AquaVac™ ERM killed a significantly higher amount of *Y. ruckeri* than plasma from sham-vaccinated control trout taken 8 weeks post-vaccination (p<0.001 and p<0.05), respectively. Plasma from fish vaccinated by the experimental bacterin killed a significantly higher number of *Y. ruckeri in vitro* compared to plasma from the AquaVac™ ERM immersion vaccinated fish (p<0.01). Twenty-six weeks post-vaccination no difference between groups could be detected with regard to the bactericidal effect of plasma but still a significantly higher bactericide effect of plasma was found when compared to PBS (p<0.05). The oral booster vaccination did not influence the bactericidal effect of plasma (Fig.5 B).

## Discussion

Immersion vaccination of salmonids against ERM has been conducted successfully for more than 30 years but the protective mechanisms activated by this type of immunization are still being debated. The involvement of specific IgM antibodies has never been clearly established but this may be due to the relatively low sensitivity of the used methods. The present work has showed that the IgM antibody titers increase significantly following immersion vaccination against ERM. Increased levels of specific IgM antibodies against *Y. ruckeri* relative to sham-vaccinated control fish were found at 8 and 12 weeks post-vaccination which correspond to the time required at 12-13°C to develop protective immunity against water borne ERM [15].

Further, it was shown that immersion vaccinated trout contained significantly less *Y. ruckeri* in the blood compared to sham-vaccinated trout 3–14 days post a bath challenge with *Y. ruckeri*, given 8 weeks post-immersion vaccination (Fig.4). This indicates that the vaccine-induced protection is, at least partly, associated with a control of bacteria in the blood. One of the protective mechanisms reducing the amount of *Y. ruckeri* in the blood of immersion vaccinated rainbow trout could be an increase of bactericidal factors in plasma. It was shown that plasma from even sham-vaccinated rainbow trout contains factors that is able to kill a significant amount of *Y. ruckeri* (*in vitro*), and that the bactericidal effect is further increased in plasma taken from immersion vaccinated trout 8 weeks post-vaccination. Plasma from ERM bacterin immersion vaccinated rainbow trout has a higher bactericidal effect than AquaVac™ ERM immersion vaccinated trout. One reason for this could be that the experimental bacterin vaccine and *Y. ruckeri* strain used for the *in vitro* experiment is the same isolate.

**Figure 3:**
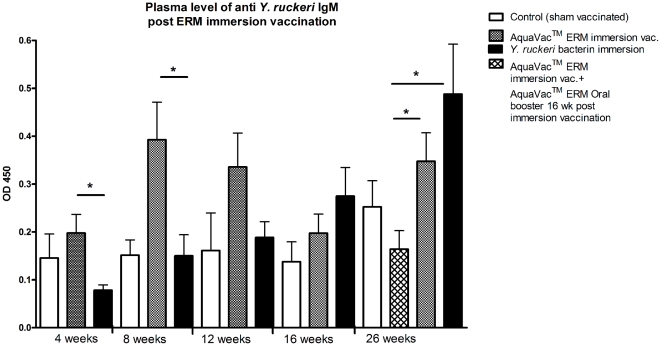
Detection of *Y. ruckeri* specific IgM antibodies in plasma post ERM immersion vaccination. The ELISA method was used to detect *Y. ruckeri* specific IgM antibodies in plasma. The antibody titer were significantly higher in immersion vaccinated groups compared to the sham vaccinated group (*P<0.05).

The vaccine induced increase in bacterial killing is probably not only due to increased antibody levels, since the bacterin vaccinated group (showing the highest *in vitro* killing of *Y. ruckeri)* showed no increase of the antibody level 8 weeks post-vaccination (Fig. 3 and 5). Further studies should elucidate if innate immune factors such as complement, lysozyme and acute phase proteins such as serum amyloid protein a (SAA) play a role. Thus, SAA transcripts are highly increased in *Y. ruckeri* infected rainbow trout [19]. SAA can bind to the outer membrane protein A (OmpA), a conserved protein among the Gram-negative bacteria belonging to Enterobacteriaceae.

**Figure 4:**
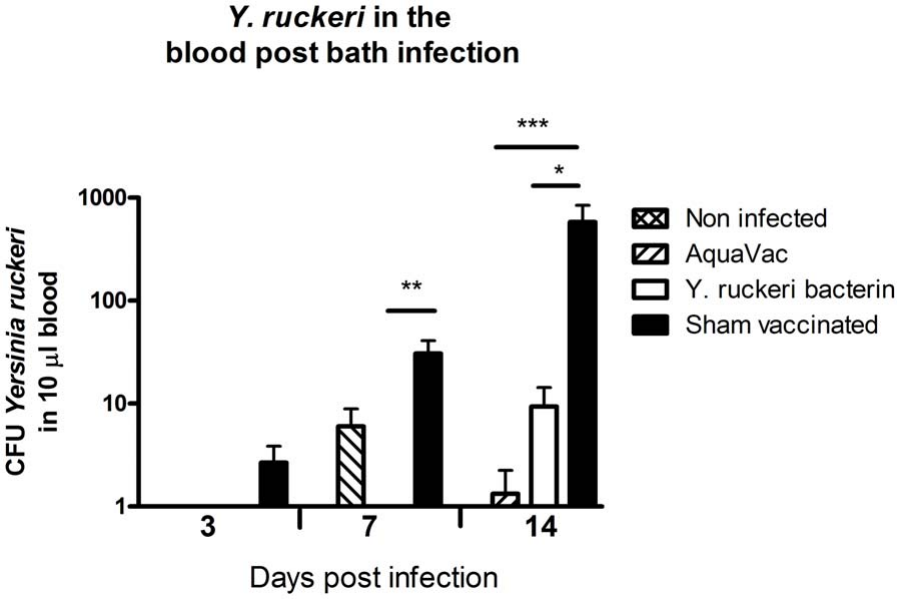
Presence of *Y. ruckeri* in blood post infection. Amount of *Y. ruckeri* in blood of bath challenged rainbow trout from each immersion vaccinated group (eight weeks post-vaccination). Blood samples (n = 5) were taken 3, 7 and 14 days post-infection. The amount of *Y. ruckeri* in blood was significantly lower in the groups of immersion vaccinated rainbow trout compared to the sham vaccinated group. Bars represent mean values + SD values. * Depicts statistical significance between vaccinated groups (*P<0.05; **P<0.01; ***P<0.001).

**Figure 5:**
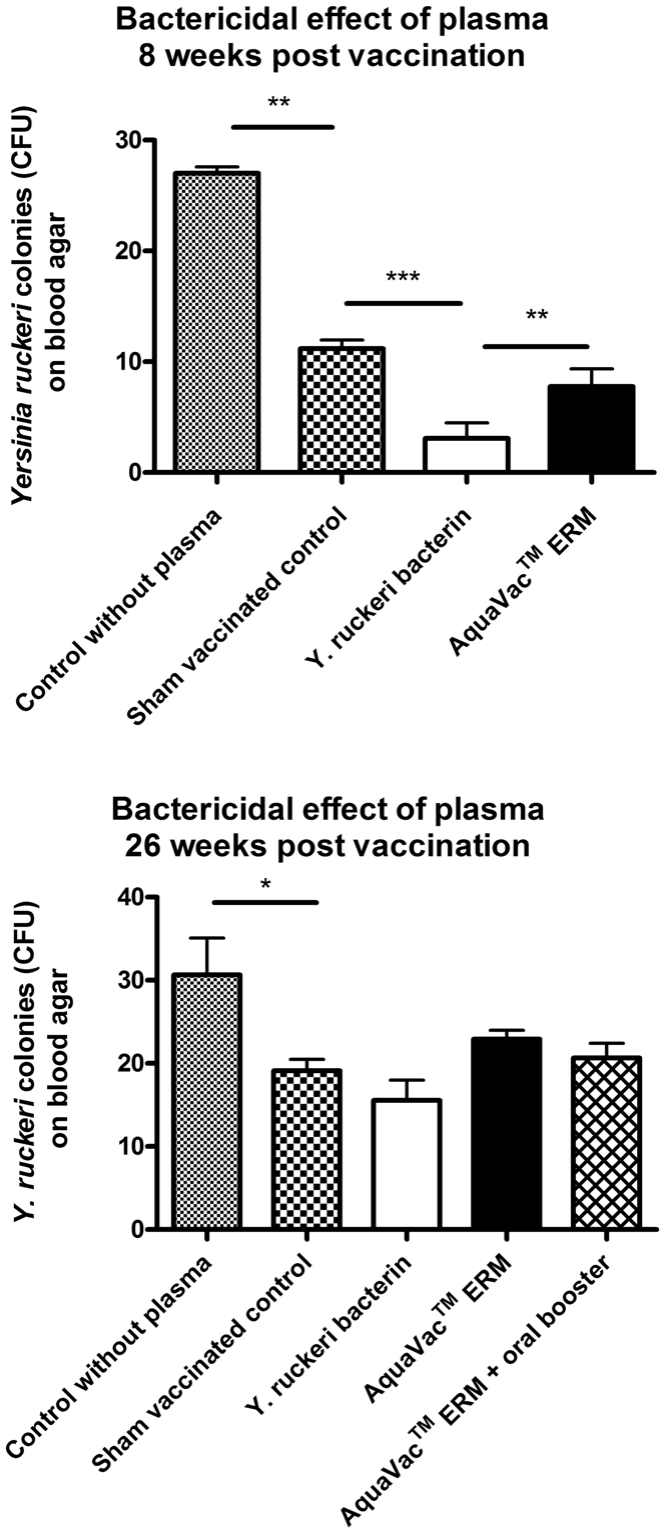
Bactericidal effect of rainbow trout plasma. The present experiment shows that plasma from sham vaccinated rainbow trout contains factors that are able to kill a significant amount of *Y. ruckeri* (A, p<0.01 and B) p<0.05. Further it is shown that plasma obtained from immersion vaccinated trout kill a significant higher amount of *Y. ruckeri* than plasma from sham-vaccinated trout. Bars represent mean values + SD values. * Depicts statistical significance between groups (*P<0.05; **P<0.01; ***P<0.001).

Although immersion vaccination with ERM bacterin is effective, it cannot prevent the appearance of outbreaks under severe stress conditions, due to exposure to a large number of bacteria disseminated by carriers and diseased fish [20]. Hence, there seems to be a discrepancy between laboratory and field tests. In particular, this regards to the duration of protection that appear to vary under farming conditions [16]. Therefore, it has been recommended that rainbow trout should be given an oral booster two to three months post immersion vaccination under field conditions in order to increase the length of immunity to cover the growth period in freshwater [21]. In the present experiment a commercial oral booster vaccine (AquaVac™ ERM Oral vet) was fed to one group of fish 16 weeks post immersion vaccination with AquaVac™ ERM as recommended by the manufacturer. The orally booster vaccinated fish showed 0% mortality during challenge with *Y. ruckeri* (Fig. 2 B). However, we were not able to show improved protection compared to fish that only received the AquaVac™ ERM immersion vaccine since they also showed 0% mortality following *Y. ruckeri* challenge (Fig. 2B). Further, the oral booster vaccination did not change the bactericidal effect of plasma and decreased the *Y. ruckeri* specific IgM titers relative to the group of fish that only received the AquaVac™ ERM immersion vaccine.

Recently it has been shown that *Y. ruckeri* has the capacity of surviving intracellularly in rainbow trout [22] which fits well with our finding of *Y. ruckeri* O1 biotype 1 in the head kidney of apparently healthy rainbow trout 6 month post i.p. infection (unpublished). Up-regulation of CD-8α transcript is previously described in ERM immersion vaccinated and protected rainbow trout fry, which indicates that activity of cytotoxic T-cells could play a role in the cellular adaptive protection mechanisms against intracellular *Y. ruckeri* stages during infection [15]. The present study has shown that antibody titers and the bactericidal effect of plasma increase in ERM immersion vaccinated rainbow trout. Moreover, this is associated with a reduced bacteremia and increased protective immunity against *Y. ruckeri* infection. Although other humoral and cellular elements may play a role in immersion vaccine induce protection of rainbow trout against ERM, the present study has indicated that specific plasma antibodies may take part in immunity towards *Y. ruckeri*.
